# Exploring multisensory integration of non-naturalistic sounds on body perception in young females with eating disorders symptomatology: a study protocol

**DOI:** 10.1186/s40337-023-00749-4

**Published:** 2023-02-27

**Authors:** Sergio Navas-León, Luis Morales Márquez, Milagrosa Sánchez-Martín, Laura Crucianelli, Nadia Bianchi-Berthouze, Mercedes Borda-Mas, Ana Tajadura-Jiménez

**Affiliations:** 1 Department of Psychology, Universidad Loyola Andalucía, Dos Hermanas, Spain; 2 Department of Neuroscience, Karolinska Institutet, Solna, Sweden; 3 UCL Interaction Centre, University College London, London, UK; 4 Department of Psychology, Universidad de Sevilla, Seville, Spain; 5 i_mBODY lab, DEI Interactive Systems Group, Department of Computer Science and Engineering, Universidad Carlos III de Madrid, Madrid, Spain

**Keywords:** Auditory feedback, Body illusions, Body image disturbance, Perception, Eating disorders, Multisensory integration, Sound

## Abstract

**Background:**

Bodily illusions can be used to investigate the experience of being in a body by manipulating the underlying processes of multisensory integration. Research suggests that people with eating disorders (EDs) may have impairments in visual, interoceptive, proprioceptive, and tactile bodily perception. Furthermore, people with EDs also show abnormalities in integrating multisensory visuo-tactile and visual-auditory signals related to the body, which may contribute to the development of body image disturbances. Visuo-auditory integration abnormalities have been observed also in people with subthreshold ED symptomatology. However, it remains unclear whether these impairments are specific to bodily signals or if they extend to any auditory signals.

**Methods:**

We will recruit 50 participants (aged 18–24; females assigned at birth) with ED symptomatology (subthreshold group) and 50 control participants. The Eating Disorder Examination Questionnaire will be administered to screen for ED symptomatology and divide the sample into two groups accordingly (control and subthreshold group using a clinical cut-off score of 2.8). The strength of both illusions will be measured implicitly with estimations of body part position and size, and explicitly with self-report questionnaires. As a secondary aim, regression analysis will be run to test the predictive role of susceptibility for both illusions on interoceptive body awareness (measured by the Multidimensional Assessment of Interoceptive Awareness Scale) and sensory-processing sensitivity (measured by the Highly Sensitive Person Scale).

**Discussion:**

Our study may contribute to our understanding of the mechanisms underlying body image disturbances. The results may pave the way for novel clinical interventions targeting early symptoms prior to the development of the disorder in young females.

**Supplementary Information:**

The online version contains supplementary material available at 10.1186/s40337-023-00749-4.

## Plain English summary

This study aims to investigate whether individuals with subthreshold eating disorder (ED) symptoms show differences in the integration of proprioceptive and auditory cues in relation to body representation, as compared to healthy controls. This will be examined by focusing on two different body parts (finger and waist) with different levels of emotional significance for individuals with EDs. Participants will be screened for ED symptoms and divided into groups accordingly. The strength of the illusions will be measured using estimations of body part position and size, as well as self-report questionnaires. Regression analysis will be used to assess the predictive role of susceptibility to the body illusions on interoceptive body awareness and sensory-processing sensitivity. The results of this study may have implications for understanding the underlying mechanisms of body image disturbances in EDs, and inform the development of clinical interventions for individuals with subthreshold EDs.

**Supplementary Information:**

The online version contains supplementary material available at 10.1186/s40337-023-00749-4.

## Background

Body image disturbance (BID) is a clinical feature commonly associated with eating disorders (EDs) [1, 2]. BID is a multidimensional construct comprising two components: an attitudinal component (i.e., negative feelings and thoughts toward the body) and a perceptual component (i.e., inability to accurately estimate body size) [2]. Traditionally, the former has played a crucial role in cognitive-behavioral therapies whereas the latter has been largely overlooked [2, 3], and thus its relevance to clinical practice remains unclear [4]. Experimental evidence suggests that people with Anorexia Nervosa (AN) and Bulimia Nervosa overestimate the body size compared to control participants [4, 5]. Furthermore, the perceptual component of BID in people with AN has been linked to poorer therapeutic outcomes, namely increased cognitive, affective, and behavioral psychopathology [6, 7]. Importantly, neurobiological studies using functional magnetic resonance imaging (fMRI) have associated impairments in the perceptual component in people with AN with structural–functional brain abnormalities in areas involved in the processing of body-related information (e.g., alterations of the precuneus or parietal cortex, among others) [7, 8]. Taken together, this body of literature suggests that the perceptual component of BID is a crucial clinical feature in EDs that requires more attention and must be targeted using specialized treatments [4, 6].

The perceptual component of BID might be a contributing factor in the onset, maintenance, and relapse of EDs, although evidence in this direction is still sparse [3]. However, recent developments in the field of multisensory integration can fill this gap by uncovering the processes underlying the perception of one’s own body, and by implications shed some light on the aetiological basis underlying perceptual disturbances in body image [9]. Ongoing research in this field suggests that our body image is not static, but it is continuously updated responding to the inputs that we receive from our body (i.e., visual, auditory, or haptic signals, among others) [9, 10]. This idea is supported by studies using multisensory body illusions, which have shown the malleability of body image, and overall mental body-representations, in response to conflicting sensory inputs [10, 11]. In broad terms, these body illusions could be defined as any “psychological phenomenon in which the perception of one's own body importantly deviates from the configuration of the physical one, e.g., in terms of size, location, or ownership” [12]. For example, as a prototypical paradigm, the Rubber Hand Illusion showed that it is possible to induce participants to perceive a rubber hand as part of their body by touching a fake hand in synchrony with the participant’s own hand, which is out of view (visual–haptic–proprioceptive integration) [11]. Inspired by this promising line of research, studies applying body illusions to investigate the perceptual component of BID on clinical conditions, including EDs, are on the rise [13].

Mussap and Salton [14] used the rubber hand illusion to evaluate the relationship between body image perception and ED symptomatology. Their results suggest that participants with ED symptomatology were significantly more susceptible to the illusion as compared to people without ED symptomatology. Thus, they concluded that body image malleability could be a risk factor for developing EDs given the significant relationship between the susceptibility to the illusion with ED symptomatology, such as bingeing and purging behaviors. Along the same line, subsequent studies corroborated and extended these findings in EDs samples. For example, Eshkevari et al. [15] found that participants with EDs experienced the illusion when the rubber hand and the participant’s own hand were touched asynchronously (i.e., a condition that does not lead to the illusion in healthy individuals) [15, 16]. In another study, Keizer et al. [17] used the ‘Full Body Illusion’ to induce people with AN to feel as if a full-body avatar was their own body. They reported that after removing visual feedback of the participant’s own body, it was possible to reduce the overestimation of participants’ own body, with results lasting at least 2 h and 45 min after the illusion was induced. Overall, these findings show an increased sensitivity to visual information about the body and a decrease attention to body somatosensory and proprioceptive information processing [15–17]. However, studies in the field reveals that perceptual impairments relevant to BID may also be non-visual, including other modalities such as haptic perception, interoception, or proprioception, among others [18, 19]. Accordingly, it is crucial to elucidate what aspect of perceptual processing of bodily information, including multisensory integration, is altered in EDs [19, 20].

Importantly, despite the growing body of literature pointing to the importance of auditory cues on body perception in healthy individuals and various clinical populations [21], there is a lack of investigation about auditory-driven body perception in EDs [22]. Along this line, Tajadura-Jiménez et al. [23] found that people's perceptions of their own body weight can change depending on the frequency spectra of their footsteps sounds (see work on the so-called ‘Footsteps Illusion’; [23]). Among the main findings, participants reported their bodies as being thinner when hearing the higher frequencies of their footsteps sound (vs. a low frequency condition) [23]. Critically, in the ED field, to the best of our knowledge, only two studies have investigated the multisensory integration of auditory signals with other sensory signals. In the first study, Chirico et al. [22] used the ‘Sound-induced Flash Illusion’, in which a single flash is presented together with several auditory beeps, to show that temporal discrimination processing of visuo-auditory stimuli was impaired in patients with AN, as compared to healthy controls. In the second study, considering the above-mentioned literature indicating a stronger influence of external sensory signals on processing bodily information, Tajadura-Jiménez et al. [24] hypothesised that participants with high-ED symptomatology and participants with AN would experience an enhanced ‘footstep illusion’ compared with those with low-ED symptomatology and control participants. This is in line with the previous hypothesis suggesting an overreliance of exteroception in people with EDs [15–17]. Contrary to expectations, they found that the AN group and the high-EDs risk group reported their body as wider/heavier in the ‘High-Frequency’ condition compared to the ‘Control-condition’. Tajadura-Jiménez et al. [24] suggested that these abnormalities in multisensory integration of proprioceptive and auditory signals may explain the difficulties in updating bodily information in light of novel and sometimes surprising sensory signals, which might potentially contribute to the development of BID in EDs. However, it still remains unclear whether these impairments are specific to natural auditory signals related to body weight, which is an emotionallysalient aspect in people with EDs, or whether they correspond to a general impairment related to the monitoring and integration of any auditory signals in relation to one’s own body [24].

Given the lack of research, the primary aim of this study is to elucidate the link between body perception and non-naturalistic auditory information in participants with subthreshold ED symptomatology, as compared to participants without ED symptomatology, using two body illusions. Our primary objective is to investigate the effect of artificial non-naturalistic sounds on the perceived size of a finger in healthy individuals. Previous research has found that this type of sound, which does not contain information on body size, can elicit changes in the perceived size of a finger in a phenomenon known as the 'auditory Pinocchio' illusion [25]. In the first study to report this illusion, it was found that when healthy participants pulled the tip of their right index finger with their left hand and an ascending pitch sound was presented concurrently, the participants both felt and judged their finger to be longer than when the pull was combined with a descending pitch or a constant pitch sound [25]. This illusion builds on the well-known association between changes in pitch and changes in height or size (for a review see [26]). This illusion focuses on a body part (finger) that is not emotionally salient, reducing therefore ‘contamination' by cognitive processes [27]. Nevertheless, given the clinical relevance of studying emotionally salient body parts as they play an essential role in BID [4], and in order to extend the aforementioned research [24], we will adapt the 'auditory Pinocchio’ illusion to a body part that is particularly salient from the emotional point of view for the majority of EDs, namely the waist. We expect that the illusion also occurs for the waist (horizontal position) as for the finger (vertical position) since besides the correspondence with height, pitch is also associated to physical size (at least in the visual domain): static high and low pitches are respectively associated to smaller and larger visual size [28], and ascending and descending pitches are respectively associated to growing and shrinking size [29]. This body of research holds the potential to provide experimental evidence supporting the development of novel therapies, which can target specifically the aforementioned distortions in the perception of sensory body signals, and ultimately BID (e.g., [30]).

For example, Mussap and Salton [14] noted, one explanation for individual differences in response to cognitive-behavior therapies may reflect differences in pre-existing levels of body-image flexibility (i.e., ability to openly perceive thoughts or sensations concerning the body without acting on or changing them) [14]. This line of reasoning is in accordance with a recent meta-analysis (N = 62) which indicated that higher body-image flexibility is associated with lower levels of body-image problems and ED symptomatology [31]. Thus, body-image flexibility may arise as a key psychological construct that may be enhanced during psychological interventions [31]. As such, both body illusions (affecting finger and waist mental representations) could be useful to potentially identify participants who will be most responsive to behavioural therapies, as those with heightened body-image flexibility. Given the lack of research, there is a need to test the association between body-image flexibility and key psychological constructs related to ED symptomatology in this area [31].

## Aim and hypothesis

### Primary exploratory aim: auditory-driven changes in body perception

The primary aim of this study is to gain an exploratory understanding of the influence on body perception of non-naturalistic auditory information in participants with subthreshold EDs symptomatology using two body illusions. The scarce literature on the topic precludes us from considering a confirmatory model. As a result, the following hypotheses are all descriptive–exploratory.

**Hypotheses 1 (H1)**: Participants with subthreshold ED symptomatology will differ from participants without ED symptomatology in their degree of body image distortion. This hypothesis is exploratory, as alternatively, both groups may show the same degree of distortion.

H1 is based on previous literature that has shown a conflicting set of results. While there is strong evidence to suggest that young females with ED symptomatology and individuals with EDs tend to overestimate their body size compared to control individuals, some evidence suggests that body image distortions are not specific to this population, implying that they can underestimate their body size [24] or even be as accurate as individuals without ED symptomatology [32].

**Hypotheses 2 (H2)**: Participants with subthreshold ED symptomatology will show stronger auditory-driven body illusions, compared to control individuals.

H2 is based on the traditional hypothesis in support of a stronger influence of external sensory signals, leading to stronger body illusions in people with and subthreshold EDs [15–17].

**Hypotheses 3 (H3)**: Participants with subthreshold ED symptomatology, as compared to without ED symptomatology, will present unusual patterns of results in the finger elongation or shrinking waist illusion, suggesting impairments in the integration of artificial auditory signals into their body image.

H3 hypothesizes that participants with subthreshold ED symptomatology, as compared to people without ED symptomatology, will present unusual patterns of results, that is, the perceived finger elongation or shrinking waist will not necessarily occur in the ascending pitch condition (or not only in that condition, as in [24]. The results will shed light on whether the alteration in the mechanisms integrating auditory signals into body image is present only to natural signals related to body weight/size (e.g., footsteps) or it is linked to any auditory signals.

**Hypotheses 4 (H4)**: Participants with subthreshold ED symptomatology, as compared to people without ED symptomatology, will experience the finger illusion but not the waist illusion, suggesting that the experience of the illusion is linked to the emotional salience of the body part rather than the nature of the auditory signals.

H4 hypothesizes that participants with subthreshold EDs symptomatology will experience the illusion for the finger (not emotionally salient body part), replicating previous research (see [25]) but not for the waist (emotionally salient body part). In that case, the experience of the illusion will not be linked to the nature of the auditory signals (natural sound vs. artificial sound), but to the emotional salience of the body part (finger vs. waist).

### Secondary exploratory aim: testing predictors

We hypothesized that the susceptibility to both body illusions, that is body image flexibility, will predict other key psychological constructs that have shown relations with ED, such as interoceptive body awareness (i.e., the ability to identify, access, understand, and respond appropriately to the patterns of internal signals) [14, 15] and sensory-processing sensitivity (i.e., the tendency to process stimuli and information more strongly and deeper than others [33]). Likewise, given the novelty of the topic, the following hypothesis are descriptive–exploratory in nature:

**Hypotheses 5 (H5)**: There is a relationship between body position/size measures and interoceptive body awareness as well as sensory-processing sensitivity.

**Hypotheses 6 (H6)**: Body position/size measures may have an effect on interoceptive body awareness and sensory-processing sensitivity.

## Material and methods

The ethics committees of the University of Loyola and of Junta de Andalucía have approved the present study, which will be performed in accordance with the ethical standards in the 1964 Declaration of Helsinki and its later amendments. All participants will provide informed consent to take part in the study.

## Participants

The experiment will be conducted individually in a quiet laboratory room [see Additional file 1, section A]. In keeping with previous literature and to facilitate the comparison and interpretation of the results because of the variability shown in related measures, the sample will comprise young adult females (sex assigned at birth) in the 18–24 age range, where the prevalence of EDs in this population is the highest [34]. However, it should be pointed out that previous evidence has reported high prevalence of EDs not only in female samples but also in other populations such as in men or gender diverse individuals [35–37]. Additionally, we decided to focus on the waist because this body part has been shown to be particularly emotionally salient for females and a reliable indicator of body mass index (BMI) [4, 38]. Participants will be recruited through public advertisements and social media posts (convenience sampling) and they will be paid or compensated with grade points for their time. To split the sample into participants with and without ED symptomatology, the Spanish Eating Disorder Examination Questionnaire (S-EDE-Q) [39]) will be administered (see Table 1 for more details). The pre-screening will allow to form two groups of similar size: once the desired participant sample size has been reached for one of the groups, only participants falling into the other group defined for the study will be invited to take part. The S-EDE-Q have demonstrated good psychometric properties in young female adults in Spain [39]. A global EDE-Q score ≥ 2.8 has been shown to provide an optimal trade-off between sensitivity and specificity when it is used for screening in primary care [40]. Available resources for participants will include psychoeducation and support for body image and eating-related concerns, provided by a team member. The availability of these resources will be specified in the informed consent. Moreover, to not assume that all individuals are cis-gender, gender identity will be assessed through an additional question.

**Table 1:** Overview of study measures and data collection timepoints

Measure	Description	Baseline	Experiment	
			1	2
Demographic data	Participants’ age. In addition, BMI will be calculated with the following formula: BMI = weight (kg)/(height (m)^2^) following the World Health Organization’s criterion [45]	X		
Information consent	Written informed consent	X		
S-EDE-Q†	To assess body EDs psychopathology. Self-administered questionnaire composed of 28 items using 7-point Likert scales ranging from 0 (not at all) to 6 points (markedly) about the previous 28 days. Four subscales are measured, including: dietary restraint (5 items), shape concerns (8 items), weight concerns (5 items), and eating concerns (5 items). For each subscale, the score ranges from 0 and 6 points. Participants will be grouped according to a global index score which is the average of the four subscale scores. As in Mond et al. [36], we will use a cut-off point ≥ 2.8 as clinically significant. Higher scores indicating a higher ED pathology. The S-EDE-Q have shown adequate internal consistency (Cronbach’s alpha ≥ .81 for all subscales), acceptable sensitivity (≥ 0.95), specificity (≥ 0.90), and positive (≥ 0.59) and negative (≥ 0.99) predictive values, and positive and significant correlation (≥ 0.70) with EDE diagnoses	X		
HSPS‡	To assess the tendency to process stimuli and information more strongly and deeply than others (sensory-processing sensitivity). Self-administered questionnaire composed of 27 items using 7-point Likert scales ranging from 0 (not at all) to 6 points (markedly). Five subscales are measured including: (1) sensitivity to overstimulation (9 items); (2) aesthetic sensitivity (6 items); (3) low sensory threshold (5 items); (4) fine psychological discrimination (4 items); (5) harm avoidance (3 items). For each subscale, the score ranges from 27 to 189 points. Higher scores indicate higher sensitivity level. A total global score will be obtained by averaging the five subscales. The HSPS have shown adequate internal consistency (Cronbach’s alpha ≥ 0.81 for all subscales) and construct validity in the adult Spanish population	X		
MAIA§	To measure interoceptive body awareness. Self-administered questionnaire composed of 32 items using 6-point Likert scales ranging from 0 (never) to 5 points (always). Eight subscales are measured, including: (1) noticing (3 items); (2) not-distracting (2 items); (3) not-worrying (2 items); (4) attention regulation (7 items); (5) emotional awareness (5 items); (6) self-regulation (4 items); (7) body listening (3 items) (8) trusting (3 items). It generates a total direct score that ranges from 0 to 160 points. Higher scores indicate higher interoceptive awareness. A total global score will be obtained by averaging the six subscales. The MAIA have shown adequate internal consistency (Cronbach’s alpha 0.64 to 0.83 for all subscales)	X		
Auditory trials	Presented in four conditions: an anchor task (20 trials); a practice task (3 trials); an experimental block (30 trials) and a questionnaire block (3 trials)		X	X
Estimation of body position (finger/waist)	To assess estimation of location or position of fingertip/knuckle (allowing to calculate the estimated finger length can be calculated) and waist width after each auditory experimental trial		X	
Confidence task	It has been found that the reliability of perception might be linked to subjective rather than objective accuracy [41]. For example, previous studies using body illusions report that confidence was not accompanied by increases in objective accuracy (i.e., [42, 43]). Therefore, to assess the reliability of their estimations, after each auditory experimental trial, the feeling of confidence with the estimation of body part position will be explicitly assessed by asking the participants: “From one to seven, how confident are you of your estimation?” (7-point Likert scale)		X	X
Body feelings questionnaire (finger/waist) [25]	To assess subjective feelings about their finger/waist after the trials (susceptibility to the illusion). Participants will respond to a questionnaire containing 7-point Likert-type response items ranging from 1 (strongly disagree) to 7 (strongly agree) (Finger: 14 items; Waist: 13 items). In addition, a range of figures representing finger/waist will be presented. Each figure shows a whole hand/trunk selectively shrunk or elongated. Participants will be asked to choose one of the figures to describe the subjective feeling of the size of their finger/waist when listening to the sound. [see Additional file 2 and 3 for more details]		X	X
Body visualizer task [44]	To assess the ability to accurately estimate body size (body size discrepancy) as well as the effects of sound feedback on perceived body size. Participants will adjust the weight related dimension of the body of a 3D avatar displayed on the screen to correspond to their perceived body size. Accuracy will be calculated according to the formula (estimated/actual body size) × 100 also-called body perception index (BPI) [45]. Specifically, a negative value represents an underestimation, whereas a positive value represents an overestimation	X		X

Body weight will be measured to the nearest 0.1 kg/% by means of a calibrated digital body fat scale (Sinocare CW 286). The Body feelings questionnaire will be directly built into Google Form and completed on a 10.1″ Android tablet (Lenovo M10)

^†^Spanish Version of the Eating Disorder Examination Questionnaire [46]

^‡^Spanish version of the Highly Sensitive Person Scale [47]

^§^The Multidimensional Assessment of Interoceptive Awareness [48]

Inclusion criteria will be: (a) normal (or corrected) auditory acuity; (b) sex: females (sex assigned at birth); (c) females within the healthy range of BMI (between 18.5 and 24.9) in order to reduce the likelihood of medical comorbidities that could affect the results (according to the World Health Organization index (WHO) [49]). Nevertheless, we recognize that weight/body mass index is not the sole predictor of potential co-occurring medical conditions. By doing so, we also avoid a potential confounding variable reported by [24]. This is in line with the criteria of healthy participants usually used in the field of eating disorders and body illusions research (e.g., [16]). Exclusion criteria: (a) lifetime history or presence of any significant brain injury, neurological condition, auditory pathology; (b) lifetime history or presence of a mental illness measured by self-report measures and screening questions; (c) lifetime history of underweight or overweight; (d) current use of psychotropic drugs (e.g. antidepressants) or intake of recreational synthetic or natural drugs; (e) inability to understand Spanish; (f) inability to provide consent; (g) out of age range; (h) pregnant status.

### Stimuli and apparatus

**Fig. 1:**
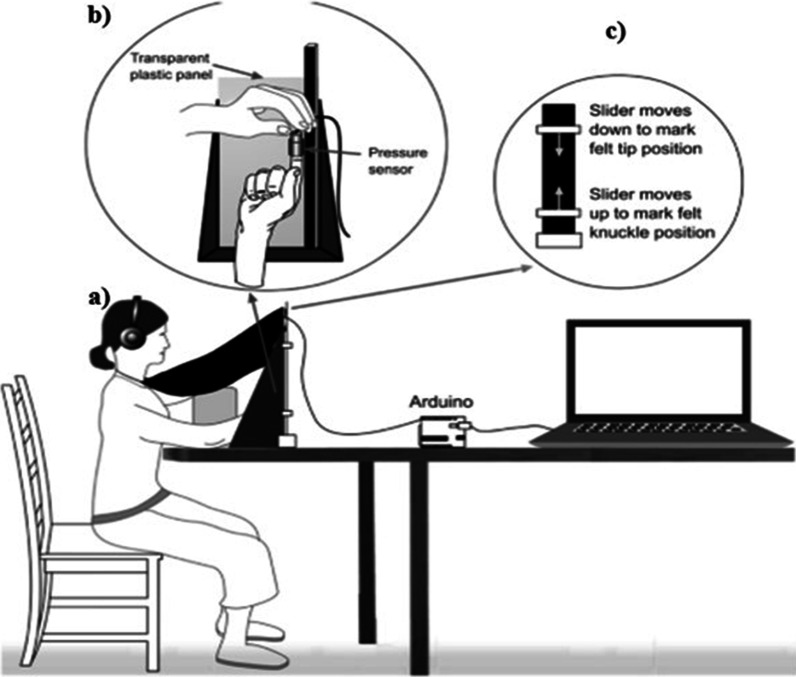
Experiment 1 experimental setup, extracted from [25]. (**a**) A vertical plastic panel (39 × 15 cm) will be attached on the bottom and right side to a vertical metal bar (2 × 2 × 50 cm). Participants will sit in front of the panel, with their right hand pressed and pulled out against it and their fingertip pointing upwards. A black cloak will be tied to the participant's neck; the other side of the cloak will be attached to the top of the panel to block the participant from seeing her own hand. (**b**) To detect the participant's finger pulling action and trigger the auditory stimulation, a force-sensitive resistor (FSR; 4 mm-diameter active sensing area) will be attached to the right index fingertip. The FSR will be connected to a computer via an Arduino microcontroller. Presentation® software (Version 18.0, Neurobehavioral Systems, Inc., Berkeley, CA, www.neurobs.com) will be employed for stimulus delivery and response recording. (**c**) A slide bar will be used to collect participants’ estimates of their fingertip and knuckle position. This apparatus consists of a ruler fixed on the right side of the metal bar, parallel to it. The ruler will be blacked out on the side facing the participant. Two horizontal clips will be mounted on the ruler, each of them with a red dot which will serve as visual points that the participant uses to mark their felt fingertip and knuckle positions. The two horizontal clips on the ruler will be initially positioned at the heights of 10 cm and 50 cm. A red fixation point will be fixed on the top centre of the plastic panel (over the cloak). Permission to reproduce the figure from: CC BY 4.0

**Fig. 2:**
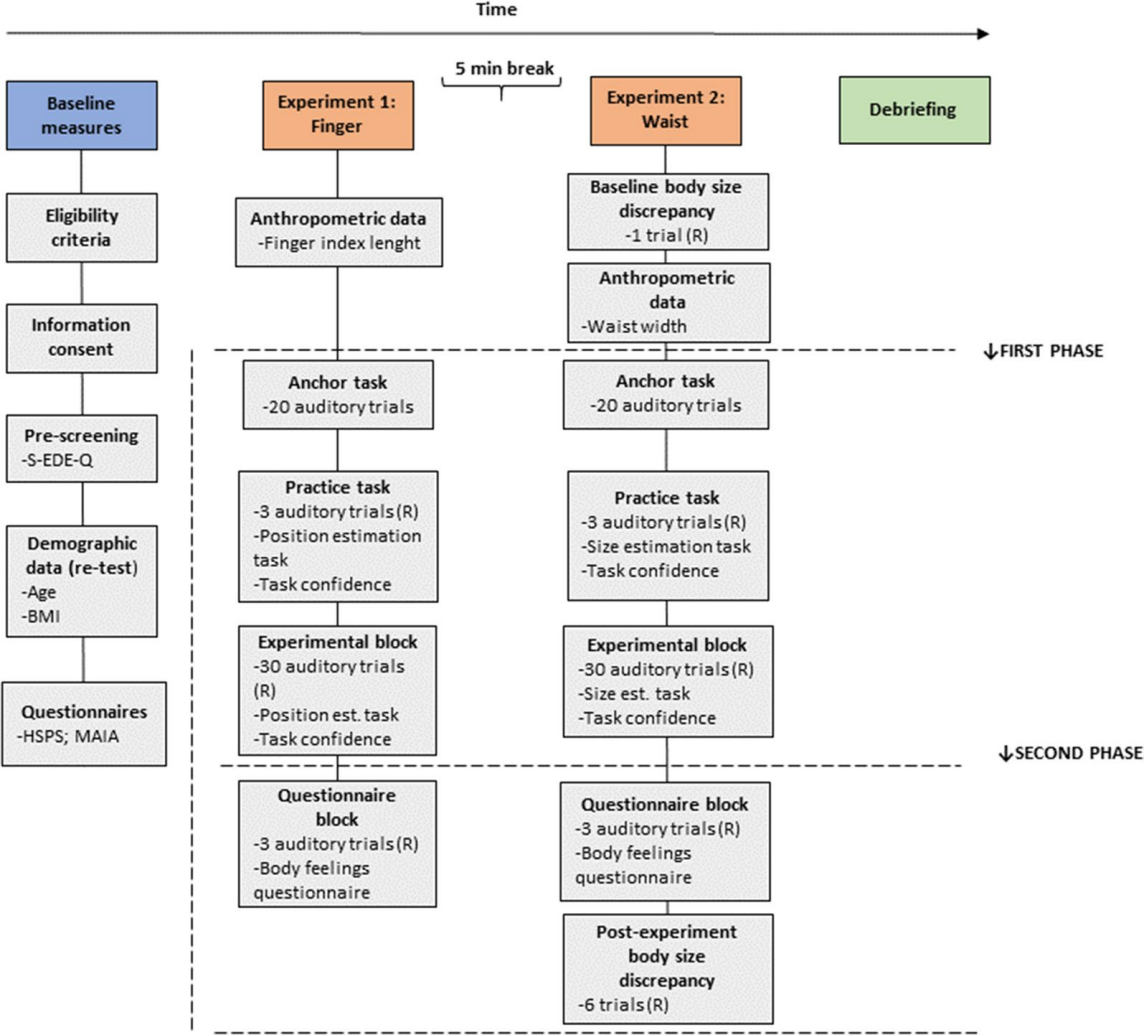
Schematic depiction of experimental procedure and timeline of events for participants with subthreshold ED symptomatology and control participants. R = Randomized order

Auditory stimuli for both Experiment 1 (finger) and 2 (waist) will be generated through Audacity software and presented via closed headphones (Sennheiser HDA300) with high passive ambient noise attenuation (> 30 dBA). The auditory stimuli will consist of pure tones (2000 ms duration, 44.1-kHz sample rate) of increasing (‘ascending’ tone: 700 to 1200 Hz), decreasing (‘descending’ tone: 700 to 200 Hz) or constant (‘constant’ tone: 700 Hz) frequency, as [25] and [50]. An additional auditory stimulus, presented in the anchor task at the beginning of the experiment, will consist of a pure tone of constant frequency (‘anchoring’ tone: 250 ms duration, 700 Hz). A 10 ms onset/offset ramp will be added to the auditory stimuli to prevent clipping. Prior to completing both experiments, participants will adjust the volume on the headphones to a comfortable level. Figures 1 and 2 show a schematic diagram and a detailed description of each experimental setup. Both experiments will be run on a HP 15S-fq4027ns Intel Core i5 laptop. Body visualizer task will be completed on a 1920 × 1080 pixel resolution computer screen (AOC e2270swn led 21.5″) (see the S1 section A). For Experiment 1, we will use a vernier calliper (DEXTER; Range: 150 mm; Graduation: 0.001″/0.02 mm) (see the S1 section B). For Experiment 2, we will use a paediatric stadiometer (ADE MZ10028-1; Range: 100 cm; Graduation: 1 mm) (see the S1 section B).

### General experimental procedure

**Fig. 3:**
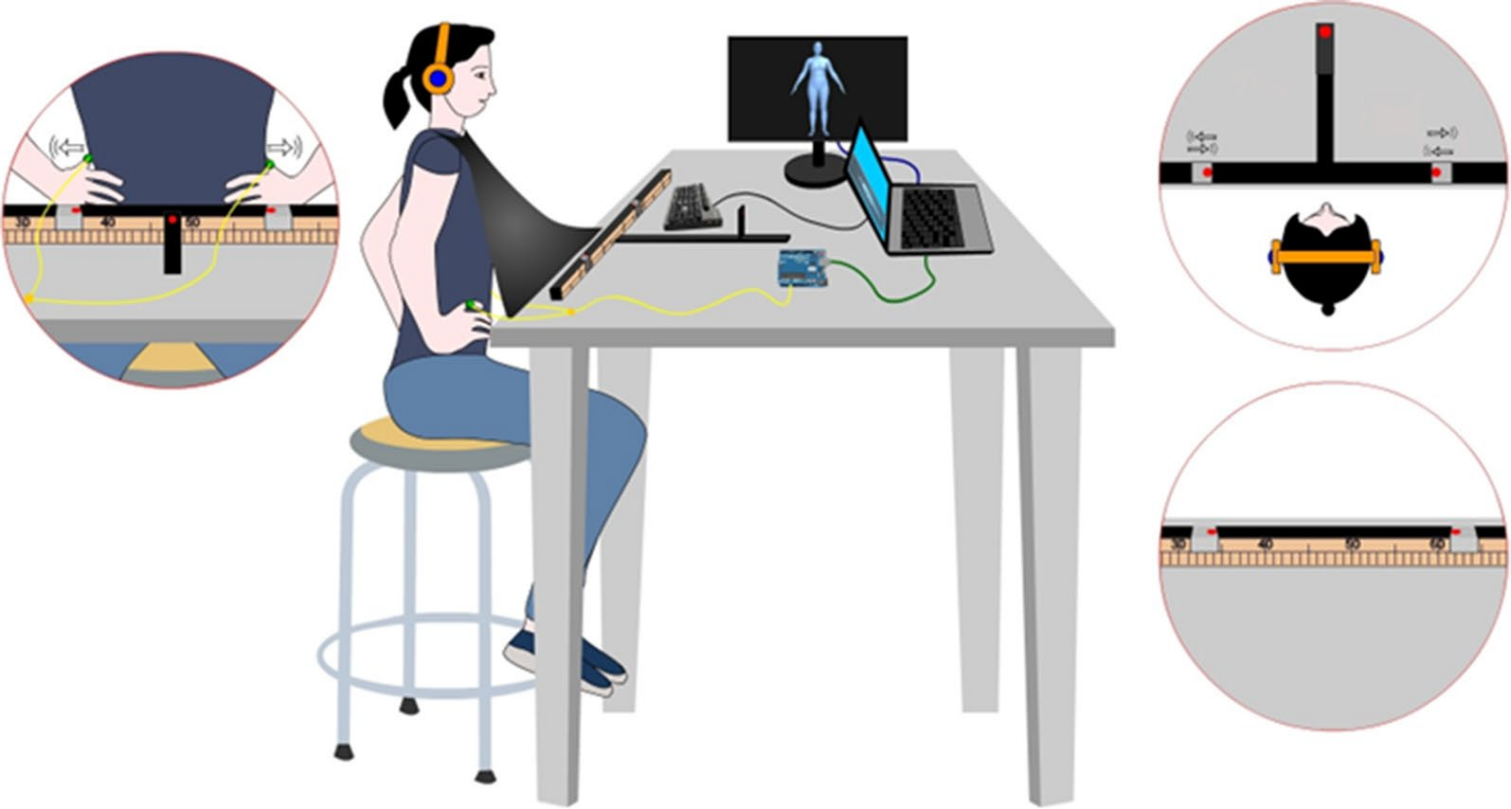
Experiment 2 experimental setup. (**a**) The participants will sit in front of a table, with both hands pressed and pulled out against their waist. A black cloak will be tied around the participant's neck; the other side of the cloak will be attached to the bottom of the table to prevent the participant seeing their body. (**b**) Similarly to Experiment 1, to detect the task of pressing and pulling out the participant's waist and activate the auditory stimulation, a force-sensitive resistor will be placed on one finger. The FSR will interface to a computer through an Arduino microcontroller. Likewise, Presentation® software version 18.0 will be used for stimulus delivery and response recording. (**c**) A horizontal ruler will be used to collect the participants' estimates of their waist width. The ruler will be located on the table. The ruler will be dark on the side facing the participant. Two horizontal metal clips will be mounted on the ruler, each with a red dot that will serve as visual points that the participant will use to mark their waist position. A red fixation point will be placed on the middle of the table

Table 1 shows an overview of study measures and data collection time points and Fig. 3 shows a schematic representation of the experimental procedure. After reading the information sheet and signing the information consent, participants will complete the S-EDE-Q. Participants eligible to take part will be invited to the lab at Loyola Andalucia University. The experimental procedures will be implemented, and all data will be collected, by a postgraduate Ph.D. researcher trained by senior members of the research team. After reading the information sheet and signing the information consent, demographic data (age and BMI) will be collected. Later, participants will complete two questionnaires related to the secondary aim (see Table 2). The Multidimensional Assessment of Interoceptive Awareness Scale (MAIA) (to assess interoceptive body awareness) and the Highly Sensitive Person Scale (HSPS) (to assess sensory-processing sensitivity). The same participants will complete both Experiments 1 and 2.

Prior to the experiments, participants will be asked to remove any jewellery from their hands and verbal and written instructions about the tasks will be given to them. Before each experiment, anthropometric data (in centimetres, thereafter ‘cm’) will be collected (finger length and waist width). Next, participants will be accommodated in the experimental setup and equipped with the pressure sensor (fixed with rubber bands) and the headphones. First, participants will be asked to complete the “anchor” task, which consists of pressing and pulling their right index fingertip with their left hand (in Experiment 1) or pressing and pulling out their waist with their index fingertips (in Experiment 2) twenty times, an action that triggers the ‘anchoring’ tone on each occasion (as in [25]). Note that during this task, participants will not be exposed to the ‘ascending’ or ‘descending’ tones. The “anchor” task uses a standard tone to facilitate participants pair the motor action and the production of a sound [25]. Having completed the “anchor” task, participants will be asked to complete a practice task, one trial for each feedback condition. Later, participants will be asked to complete the experimental block.

The procedure and materials of each of the experimental blocks is described in next sections and in Figs. 1 and 2. In brief, in the experimental block each experimental tone (‘ascending’, ‘descending’, ‘constant’), will be presented ten times. The order in which the tones are presented will be counterbalanced across participants following a within-subject design. After the experimental block is completed, participants will be asked to repeat the task of pulling their right finger (Experiment 1) or waist sides (Experiment 2) while listening to a tone for three more trials, one trial for each feedback condition, with the presentation order randomized across participants. Participants will be asked to complete a body feelings questionnaire after each feedback condition (questionnaire block) [see Additional file 2 and 3 for more details]. Additionally, for Experiment 2, a baseline and post-experiment body size discrepancy measure will be collected. The full experimental procedure will take approximately 120 min with a 5-min rest interval between both experiments. After completing the study, a debriefing session will be carried out.

#### Experiment 1 procedure: Finger

First, the length of the index finger of the participant’s right hand will be measured by the experimenter using a vernier calliper from the proximal crease at the base of the finger to the tip of the finger. Next, participants will be required to complete an "anchor" task. In this task, participants will be asked to press and pull out their right index fingertip with their left hand twenty times, an action that will trigger the ‘anchoring’ tone on each occasion. After completing the anchor task, participants will complete a practice task, one trial for each feedback condition.

In each practice trial, participants are required to stare straight at the red fixation point and complete the simple action of pressing and pulling their right index fingertip with their left hand while keeping the right index finger in a fixed position (straight against the plastic panel and metal bar, see Fig. 1). One of the three experimental tones ('ascending,' 'descending,' or 'constant') is triggered by the pressing/pulling action. Participants are instructed to continue pressing and pulling their fingers until the sound is no longer audible and, once the sound stops, to relax their left hand while continuing to grasp their right finger with the left hand. They will be then asked to estimate the position of their right fingertip and knuckle by having the experimenter adjust the two visible points on the ruler clips. This adjustment will be done as follows: First, the experimenter will move the top clip downwards at a continuous speed until the participant indicates that the visual point on the clip has reached the fingertip position with a "*Stop*" signal. Following a similar procedure, the experimenter will move the bottom clip upwards at a continuous speed until the participant indicates that the visual point on the clip has reached the knuckle position with a "*Stop*" signal.

Additionally, for each estimation (fingertip and knuckle), the feeling of confidence with the estimations made will be assessed.

The experimenter will use the measurements on the back of the ruler to record the fingertip and knuckle positions, rounding to the nearest 0.5 cm. After each trial, the clips are relocated. After completing the practice task, the experimental block will begin. Participants will repeat the task for thirty trials. Each experimental tone will be presented ten times. The order of trials will be randomized across participants. Participants will be given the opportunity to rest after each 10 trials.

After the experimental block, participants will be asked to repeat the task for three more trials (one for each sound condition, their order randomized across participants), in order to collect self-report measures (questionnaire block). After each trial, participants will complete a questionnaire that assesses their subjective experience during the task [see Additional file 2].

#### Experiment 2 Procedure: Waist

First, to assess the degree of baseline body size discrepancy participants will complete the Body Visualizer task. The initial ‘weight’ of the avatar will be set to match the participant’s ± 25%. Participants will be instructed to adjust the avatar's body's 'weight' dimension. Whether the initial weight was + 25% or − 25% will be counterbalanced over two repeats, one after the other, and which together allows calculating the degree of body size discrepancy for each condition, by averaging the two responses. Later, the experimenter will measure the width of the participant’s waist using a paediatric stadiometer. Specifically, the location of the waist will be “the minimum horizontal circumference around the body at waist height” in line with the ‘Standard Terminology Relating to Body Dimensions for Apparel Sizing’ [51].

Subsequently, participants will be first required to complete an "anchor" task. In this task, participants will be asked to press and pull out their waist twenty times, an action that will trigger the ‘anchoring’ tone on each occasion. After completing the anchor task, a practice task will be presented, one trial for each feedback condition.

In each practice trial, participants are required to look straight at the red fixation point in the middle of the table and complete the simple action of pressing and pulling their waist with both hands while keeping them fixed to their waist (see Fig. 2. One of the three experimental tones ('ascending,' 'descending,' or 'constant') is triggered by the pressing and pulling action. Participants are instructed to continue pressing and pulling their hands until the sound is no longer audible and, once the sound stops, to relax both hands (while keeping touching their waist). They will then be asked to determine the width of their waist by having the experimenter adjust the two visible points on the ruler clips. As a paradigmatic example, this adjustment will be done as follows: as in Keizer et al. [18], first, the experimenter will move both clips closer together at a continuous speed until the participant indicates that the clips have reached the perceived waist width position with a "Stop" signal. Similarly, the starting point of the aperture width (A) will be set at A = 2.0 × W (W = waist width in cm). This will be repeated while the clips are closed, and while the experimenter pressed and pulled them apart. The initial position of the clips will be counterbalanced across the trials. For both conditions we will collect waist width estimation measurements. Similarly, for each condition, the feeling of confidence with the estimation will be assessed and an average score will be obtained.

As in the finger experiment, the experimenter will use the measurements on the back of the ruler to record the position of the clips, rounding to the nearest 0.5 cm. After completing the practice task, the experimental block will begin. They will repeat the task for thirty trials. Each experimental tone will be presented ten times. The order of the trials will be randomized among participants. Participants will have an opportunity to rest after each 10 trials.

After the experimental block, participants will be asked to repeat the task for three more trials (one for each sound condition, their order randomized across participants) in order to collect self-report measures (questionnaire block). After each trial, participants will complete a questionnaire that assesses their subjective experience during the task [see the Additional file 3]. In addition, to assess the degree of post-experiment body size discrepancy, participants will complete the Body Visualizer task. They will repeat the task for three trials, one trial for each feedback condition. The order of the trials will be randomized among participants.

## Data analysis plan

IBM SPSS Statistics version 28.0 will be used for data inspection, descriptive and inferential statistics. All the analysis will be done by one of the investigators and it will be cross-checked by a second investigator. For transparency purposes, processed data derived from this study will be available in our OSF project.

### Data inspection

To ensure methodological rigour, prior to running the main analyses, data will be screened for normality through Shapiro–Wilk tests, QQ plots, histograms and values of skewness and kurtosis. We will examine the generated dataset to ascertain any outliers, aberrant response patterns (e.g., deviate from the typical responses) and patterns of missing data. For data related to questionnaires, we plan to use Full Information Maximum Likelihood [52]. For data related to body position/size, we plan to use average imputation as in [25].

### Data management

#### Estimation of body position/size and confidence task

For both experiments, data from the 10 repetitions for each condition will be averaged. As a rule of thumb, for participants with one trial per condition excluded, data for that trial will be replaced by the mean value of the other nine trials for that condition (as in [25]). Conversely, participants with more than one trial per condition excluded, will be excluded from all analyses. Overall, data from trials exceeding three standard deviations from the mean group value will be excluded.

For Experiment 1, we will collect measures for estimated knuckle position and fingertip position, as well as level of confidence with each estimation. In addition, for each trial, the estimated knuckle position will be subtracted from the estimated fingertip position in order to calculate the estimated finger length (as in [25]). For Experiment 2, we will collect waist width estimations, as well as level of confidence with each estimation. For each trial, we will estimate the perceived waist width by averaging the two responses given by the participant (i.e., for the opening and closing aperture).

For each participant a total of 12 summary scores will be calculated [(knuckle position + fingertip position + finger length + waist width limits) × (3 feedback conditions: ‘ascendent’, ‘constant’, ‘descendent’)]. Similarly, a total of 12 summary scores for the confidence task will be calculated [(confidence knuckle position + confidence fingertip position + confidence finger length + confidence perceived waist width limits) × (3 feedback conditions: ‘ascendent’, ‘constant’, ‘descendent’)].

Data from Presentation® software (Version 18.0, Neurobehavioral Systems, Inc., Berkeley, CA, www.neurobs.com) will be recorded into a file in UTF-8 format. The file will be organized and imported into Microsoft Excel 2021 and subsequently into IBM SPSS Statistics version 28.0. Data generated associated with the anchor tasks and practice tasks will be disregarded.

#### Body feelings questionnaire

Per participant, a total of six questionnaires will be filled [(Experiment 1 + Experiment 2) × (3 feedback conditions: ‘ascendent’, ‘constant’, ‘descendent’)]. We will use Google form for data collections and, to ensure the confidentiality and security of participant information, data will be moved to a secure server linked to the university, with encryption and other security measures in place. All participant-related data will be stored by using research identification numbers/codes that uniquely identify each user and will furthermore be password-protected to ensure the privacy of the participants. The sheets will be organized and imported into Microsoft Excel 2021 and subsequently into the statistical software IBM SPSS Statistics version 28.0. By default, we don’t expect missing values.

#### Body visualizer task

The degree of body size discrepancy will be hand-coded by the experimenter into a data sheet. Per participant, a total of four summary estimations will be calculated [(baseline body size discrepancy) + (post-experiment body size discrepancy × 3 feedback conditions: ‘ascendent’, ‘constant’, ‘descendent’)] according to the BPI index (see Table 1 for more details).

### Descriptive statistics

For the final sample, data will be expressed and pulled as means and standard deviations for parametric data whereas median and interquartile range will be used for non-parametric data. Additionally, skewness, and kurtosis for the continuous variables and frequency counts and percentages for the categorical variables will be reported. Cronbach’s α for each questionnaire will be reported for each questionnaire.

### Sample size considerations

As these analyses are intended to generate future confirmatory hypotheses, no sample size calculation was conducted. As Jones et al. [53] noted, sample size calculation may be of little value in early exploratory studies where there is a lack of previous research. In an exploratory study, the primary focus is on generating new research questions, identifying patterns and trends, and identifying potential areas for further research, rather than on testing a specific confirmatory hypothesis. As such, the sample size in an exploratory study may not be determined based on statistical power or precision and could be relatively small. Instead, the sample size may be based on the need to obtain a sufficient amount of data to allow for the exploration and identification of meaningful patterns and trends.

In this regard, a total of 100 participants (50 participants in each group) will allow us to gain an exploratory understanding between body perception and non-naturalistic auditory information in participants with subthreshold ED symptomatology. This sample size is the minimum recommended by Brysbaert [54] to seek differences between groups and is also between 30 and 150 participants, that is the sample size usually recommended in exploratory study designs with non-probability sampling [55]. To this end, and based on norms for the Spanish version of the Eating Disorders Examination Questionnaire (S-EDE-Q), we will screen approximately at least 450 young adult females [39].

Due to the limited sample size, we acknowledge that the statistical power for each of the hypotheses proposed in this study to detect differences between groups might not be sufficient according to the traditional standards in experimental psychology (effect size = 0.4, power = 0.80 and alpha value = 0.05) [54]. Therefore, the results should be taken with caution and should be contextualized within an exploratory framework. In case of meaningful results, the present exploratory study will provide the necessary foundation for a formal sample size calculation for a future confirmatory larger-scale study.

### Statistical analyses

#### Primary aim: auditory-driven changes in body perception Baseline measures

To determine differences between the groups, analyses will be performed with a Student's t-test paired comparison (for normal distribution) or Wilcoxon tests (for non-normal distribution) for ED symptomatology (EDE-Q), interoceptive body awareness (MAIA), sensory-processing sensitivity (HSPS) and body size discrepancy (Body visualizer tool).

#### Experiment 1 and Experiment 2

For the estimations of body part position/size, confidence task and items from the body feelings questionnaires we will follow a 2 × 3 ANOVA within-between interaction repeated-measures design (see Table 2). Specifically, we will use non-parametric ANOVAs on aligned rank (ART) data, suitable for ordinal data, using the R package ARTool [56]. The ANOVAs will involve a group factor with 2 levels (subthreshold EDs and controls) and a feedback condition factor with 3 levels (‘ascendent’, ‘constant’, ‘descendent’). Prior the analysis, assumptions of independence, normality and sphericity will be checked. As in [24], we will include the degree of body size discrepancy as covariate in case of significant baseline differences. Appropriate post-hoc tests will be conducted in case of significant differences.

**Table 2:** ANOVA 2 × 3 design

Group	Feedback condition		
	Ascendent	Constant	Descendent
Subthreshold EDs	Subthreshold EDs–Ascendent	Subthreshold EDs–Constant	Subthreshold EDs–Descendent
Controls	Controls–Ascendent	Controls–Constant	Controls–Descendent

For body size discrepancy, we will follow a 2 × 3 ANOVA within-between interaction repeated-measures design (see Table 3). The ANOVAs will involve a group factor with 2 levels (subthreshold EDs and controls), a time factor with two levels (baseline and post-experiment) and a feedback condition factor with 3 levels (‘ascendent’, ‘constant’, ‘descendent’).

**Table 3:** ANOVA 2 × 2 × 3 design

Group	Baseline	Experiment		
		Feedback condition		
		Ascendent	Constant	Descendent
Subthreshold EDs	Subthreshold EDs Baseline	Subthreshold EDs Post-experiment Ascendent	Subthreshold EDs Post-experiment Constant	Subthreshold EDs Post-experiment Descendent
Controls	Controls Baseline	Controls Post-experiment Ascendent	Controls Post-experiment Constant	Controls Post-experiment Descendent

The degree of body size discrepancy as covariate will be included in case of significant baseline differences. Similarly, assumptions of independence, normality and sphericity will be checked and post-hoc tests will be conducted if appropriate.

Following a similar approach as in [24], the proposed analysis will be based on the division of the controls vs. sub threshold group based on scores falling above or below the cut off of 2.8 on the EDE-Q. A complementary, secondary correlational analysis will be performed between estimation of body position/size measures and EDE-Q scores considering the entire sample.

#### Secondary aim: testing predictors

Only outcomes showing significant bivariate correlations with the predictor will be included in the final multivariate logistic regression model and these selected variables will be forced into the model using enter method. For these bivariate correlations, we will use Spearman’s Rho for non-parametric data and Pearson’s r product-moment correlation coefficient for parametric data. Prior to the analysis we will check for multicollinearity, homoscedasticity, normality of the residuals as well as independence/variance of the residuals and any bias influencing the model. If these assumptions do not hold, then non-linear regression analysis will be used.

For each experiment, we will conduct multiple linear regression analysis on the entire sample to explore the predictive role of body-image flexibility (estimation of body position/size measures as inputs) on different outcomes such as interoceptive body awareness (MAIA) and sensory-processing sensitivity (HHSPS).

## Conclusions

Accumulated evidence points towards the need for a better understanding of what aspect of perceptual processing of bodily information, including multisensory integration, is altered in EDs [19, 20]. Addressing this issue holds the potential to lay the ground for novel therapeutic interventions targeting the perceptual component of BID, which has been identified as a core feature of ED symptomatology [5]. To target this outstanding question, the present exploratory study will provide critical knowledge about the link between body perception and non-naturalistic auditory information in young normal weight females with ED symptomatology using two body illusions with different levels of emotional saliency for people EDs. The potential significance and impact of this study are noteworthy since it is still unclear whether the abnormalities in integrating auditory signals are specific to natural auditory signals related to body size or whether they extend to a general impairment related to any auditory signals. Furthermore, given that EDs treatments and body image-directed interventions still yield modest therapeutic outcomes [57–59], it is crucial to be able to identify possible predictors of individuals at high risk of developing EDs, who may potentially be targeted by specific preventive intervention such as body-image flexibility. An early identification of such young females might be the best way to reduce the incidence of EDs, as we know that EDs have a very negative prognosis and high level of relapses. From a clinical perspective, this study might pave the wave for the development of novel therapeutic approaches for BID in EDs.

An important direction for future research is to include more diverse populations with samples consisting of males and/or groups based on gender identity and sexual orientation. This would increase the external validity of the findings and allow for a more thorough examination of the factors that may influence body concerns in different populations. Additionally, future research could investigate the role of muscularity-oriented goals in body concerns, as well as any potential differences in the effects of thinness-focused goals across different populations [35–37]. Overall, this line of research has the potential to provide valuable insights into the factors that contribute to body concerns and the ways in which they may vary across different populations.

## Supplementary Information

**Additional file 1**. Experimental setup. Section A: Laboratory room. Section B: Anthropometric instruments: Vernier caliper and pediatric stadiometer.

**Additional file 2**. Experiment 1: Finger. Body feelings questionnaire. Section A: Spanish version. Section B: English version.

**Additional file 3**. Experiment 2: Waist. Body feelings questionnaire. Section A: Spanish version. Section B: English version.

## Data Availability

The datasets generated and/or analysed during the current study will be available in the Open Science Framework.

## Abbreviations

AN: Anorexia Nervosa
BMI: Body Mass Index
BID: Body Image Disturbance
EDs: Eating Disorders
EDE-Q: Eating Disorder Examination Questionnaire
MAIA: Multidimensional Assessment of Interoceptive Awareness
HSPS: Highly Sensitive Person Scale

## Declarations

### Study status

Pilot studies and recruitment/data collection began in June 2022.

## References

[1] American Psychiatric Association. Diagnostic and statistical manual of mental disorders. 5th ed. 2013.

[2] Cash TF, Smolak L. Body image. A handbook of science, practice and prevention. Body Image A Handb Sci Pract Prev. 2011.

[3] Glashouwer KA, van der Veer RML, Adipatria F, de Jong PJ, Vocks S (2019). The role of body image disturbance in the onset, maintenance, and relapse of anorexia nervosa: a systematic review. Clin Psychol Rev.

[4] Mölbert SC, Klein L, Thaler A, Mohler BJ, Brozzo C, Martus P (2017). Depictive and metric body size estimation in anorexia nervosa and bulimia nervosa: a systematic review and meta-analysis. Clin Psychol Rev.

[5] Cornelissen P, Tovée MJ (2021). Targeting body image in eating disorders. Curr Opin Psychol.

[6] Brown TA, Shott ME, Frank GKW (2021). Body size overestimation in anorexia nervosa: contributions of cognitive, affective, tactile and visual information. Psychiatry Res.

[7] Hagman J, Gardner RM, Brown DL, Gralla J, Fier JM, Frank GKW (2015). Body size overestimation and its association with body mass index, body dissatisfaction, and drive for thinness in anorexia nervosa. Eat Weight Disord.

[8] Gaudio S, Quattrocchi CC (2012). Neural basis of a multidimensional model of body image distortion in anorexia nervosa. Neurosci Biobehav Rev.

[9] Dakanalis A, Manzoni GM, Castelnuovo G, Riva G, Clerici M (2017). Towards novel paradigms for treating dysfunctional bodily experience in eating disorders. Eat Weight Disord.

[10] Azanõn E, Tamè L, Maravita A, Linkenauger SA, Ferrè ER, Tajadura-Jiménez A (2016). Multimodal contributions to body representation. Multisens Res.

[11] Botvinick M, Cohen J (1998). Rubber hands ‘feel’ touch that eyes see. Nature.

[12] Kilteni K, Maselli A, Kording KP, Slater M (2015). Over my fake body: Body ownership illusions for studying the multisensory basis of own-body perception. Front Hum Neurosci.

[13] Matamala-Gomez M, Maselli A, Malighetti C, Realdon O, Mantovani F, Riva G. Body illusions for mental health: a systematic review. Psyarxiv [Preprint]. 2021 arXiv:2106.06638 10.3390/jcm10010139 PMC7796179 33401596

[14] Mussap AJ, Salton N (2006). A “rubber-hand” illusion reveals a relationship between perceptual body image and unhealthy body change. J Health Psychol.

[15] Eshkevari E, Rieger E, Longo MR, Haggard P, Treasure J (2012). Increased plasticity of the bodily self in eating disorders. Psychol Med.

[16] Eshkevari E, Rieger E, Longo MR, Haggard P, Treasure J (2014). Persistent body image disturbance following recovery from eating disorders. Int J Eat Disord.

[17] Keizer A, Van Elburg A, Helms R, Dijkerman HC (2016). A virtual reality full body illusion improves body image disturbance in anorexia nervosa. PLoS ONE.

[18] Keizer A, Smeets MAM, Dijkerman HC, Uzunbajakau SA, van Elburg A, Postma A (2013). Too fat to fit through the door: first evidence for disturbed body-scaled action in anorexia nervosa during locomotion. PLoS ONE.

[19] Teaford M, McMurray MS, Billock V, Filipkowski M, Smart LJ (2021). The somatosensory system in anorexia nervosa: a scoping review. J Exp Psychopathol.

[20] Malighetti C, Gaudio S, Di Lernia D, Matamala-Gómez M, Riva G. Altered inner body perception in anorexia and bulimia nervosa: a systematic review. Psyarxiv [Preprint]. 2020. Available from: https://psyarxiv.com/2x4em/10.3390/jcm11237134 PMC9737310 36498708

[21] Stanton TR, Spence C (2020). The influence of auditory cues on bodily and movement perception. Front Psychol.

[22] Chirico A, Malighetti C, Serino S, Cipresso P, Pedroli E, Tuena C (2019). Towards an advancement of multisensory integration deficits in anorexia nervosa: exploring temporal discrimination processing of visuo-auditory stimuli. Annu Rev Cyber Therapy Telemed..

[23] Tajadura-Jiménez A, Basia M, Deroy O, Fairhurst M, Marquardt N, Bianchi-Berthouze N (2015). As light as your footsteps: altering walking sounds to change perceived body weight, emotional state and gait. Conf Hum Factors Comput Syst Proc.

[24] Tajadura-Jiménez A, Crucianelli L, Zheng R, Cheng C, Ley-Flores J, Borda-Más M, Fotopoulou A (2022). Body weight distortions in an auditory-driven body illusion in subclinical and clinical eating disorders. Sci Rep.

[25] Tajadura-Jiménez A, Vakali M, Fairhurst MT, Mandrigin A, Bianchi-Berthouze N, Deroy O (2017). Contingent sounds change the mental representation of one’s finger length. Sci Rep.

[26] Deroy O, Fernandez-Prieto I, Navarra J, Spence C, Hubbard TL (2018). Unraveling the paradox of spatial pitch. Spatial biases in perception and cognition.

[27] Cornelissen KK, Cornelissen PL, Hancock PJB, Tovée MJ (2016). Fixation patterns, not clinical diagnosis, predict body size over-estimation in eating disordered females and healthy controls. Int J Eat Disord.

[28] Evans KK, Treisman A (2010). Natural cross-modal mappings between visual and auditory features. J Vis.

[29] Eitan Z, Schupak A, Gotler A, Marks LE (2014). Lower pitch is larger, yet falling pitches shrink: interaction of pitch change and size change in speeded discrimination. Exp Psy.

[30] Riva G, Serino S, Di Lernia D, Pavone EF, Dakanalis A (2017). Embodied medicine: Mens sana in corpore virtuale sano. Front Hum Neurosci.

[31] Linardon J, Anderson C, Messer M, Rodgers RF, Fuller-Tyszkiewicz M (2021). Body image flexibility and its correlates: a meta-analysis. Body Image.

[32] Ergüney Okumuş FE, Sertel Berk HÖ, Yücel B (2019). Body image, depression and eating behaviour: a comparative study in eating disordered females and healthy controls. Psychiatry Clin Psychopharmacol.

[33] Metral M, Gonthier C, Luyat M, Guerraz M (2017). Body schema illusions: a study of the link between the rubber hand and kinesthetic mirror illusions through individual differences. Biomed Res Int.

[34] Touchette E, Henegar A, Godart NT, Pryor L, Falissard B, Tremblay RE (2011). Subclinical eating disorders and their comorbidity with mood and anxiety disorders in adolescent girls. Psychiatry Res.

[35] Knight Night R, Carey M, Jenkinson P, Preston C (2022). The impact of sexual orientation on how men experience disordered eating and drive for muscularity. J Gay Lesbian Ment Health..

[36] Murray SB, Nagata JM, Griffiths S, Calzo JP, Brown TA, Mitchison D (2017). The enigma of male eating disorders: a critical review and synthesis. Clin Psychol Rev.

[37] Meneguzzo P, Collantoni E, Meregalli V, Favaro A, Tenconi E (2022). Addressing weight bias in the cisgender population: differences between sexual orientations. Nutrients.

[38] Irvine KR, McCarty K, Pollet TV, Cornelissen KK, Tovée MJ, Cornelissen PL (2019). The visual cues that drive the self-assessment of body size: dissociation between fixation patterns and the key areas of the body for accurate judgement. Body Image.

[39] Peláez-Fernández MA, Labrador FJ, Raich RM (2013). Datos normativos de la versión española del eating disorders examination questionnaire (S-EDE-Q). Psicothema.

[40] Mond JM, Myers TC, Crosby RD, Hay PJ, Rodgers B, Morgan JF (2008). Screening for eating disorders in primary care: EDE-Q versus SCOFF. Behav Res Ther.

[41] Fairhurst MT, Travers E, Hayward V, Deroy O (2018). Confidence is higher in touch than in vision in cases of perceptual ambiguity. Sci Rep.

[42] De Coster L, Sánchez-Herrero P, López-Moreno J, Tajadura-Jiménez A (2021). The perceived match between observed and own bodies, but not its accuracy, is influenced by movement dynamics and clothing cues. Front Hum Neurosci.

[43] Ley-Flores J, Alshami E, Singh A, Bevilacqua F, Bianchi-Berthouze N, Deroy O (2022). Effects of pitch and musical sounds on body-representations when moving with sound. Sci Rep.

[44] Perceiving Systems MPI IS. 2011. Body Visualizer. MPI IS Perceiving Systems Department, Copyright Max Planck Gesellschaft. Retrieved July 31, 2021, from http://bodyvisualizer.com

[45] Slade PD, Russell GFM (1973). Awareness of body dimensions in anorexia nervosa: cross-sectional and longitudinal studies. Psychol Med.

[46] Peláez-Fernández MA, Labrador FJ, Raich RM (2012). Validation of Eating Disorder Examination Questionnaire (EDE-Q)—Spanish Version—for screening eating disorders. Span J Psychol.

[47] Chacón A, Pérez-Chacón M, Borda-Mas M, Avargues-Navarro ML, López-Jiménez AM (2021). Cross-cultural adaptation and validation of the highly sensitive person scale to the Adult Spanish Population (HSPS-S). Psychol Res Behav Manag.

[48] Mehling WE, Acree M, Stewart A, Silas J, Jones A (2018). The multidimensional assessment of interoceptive awareness, version 2 (MAIA-2). PLoS ONE.

[49] World Health Organization; 2022 [cited 2022 May 5]. Available from: https://www.euro.who.int/en/health-topics/disease-prevention/nutrition/a-healthy-lifestyle/body-mass-index-bmi

[50] Nava E, Tajadura-Jiménez A (2020). Auditory-induced body distortions in children and adults. Sci Rep.

[51] American Society for Testing and Materials. (2014). Standard Terminology Relating to Body Dimensions for Apparel Sizing (ASTM D5219–09). https://www.astm.org/d5219-15.html

[52] Arbuckle JL, Marcoulides GA, Schumacker RE (1996). Full information estimation in the presence of incomplete data. Advanced structural equation modeling.

[53] Jones S, Carley S, Harrison M (2003). An introduction to power and sample size estimation. Emerg Med J.

[54] Brysbaert M (2019). How many participants do we have to include in properly powered experiments? A tutorial of power analysis with reference tables. J Cognit.

[55] Daniel, J. (2012). Choosing the size of the sample. Sampling essentials: practical guidelines for making sampling choices, 2455, 236–53

[56] Wobbrock JO, Findlater L, Gergle D, Higgins JJ (2011). The aligned rank transform for nonparametric factorial analyses using only ANOVA procedures. Conf Hum Factors Comput Syst Proc.

[57] Carey M, Preston C (2019). Investigating the components of body image disturbance within eating disorders. Front psychiatry.

[58] Solmi M, Wade TD, Byrne S, Del Giovane C, Fairburn CG, Ostinelli EG (2021). Comparative efficacy and acceptability of psychological interventions for the treatment of adult outpatients with anorexia nervosa: a systematic review and network meta-analysis. The Lancet Psychiatry.

[59] Ziser K, Mölbert SC, Stuber F, Giel KE, Zipfel S, Junne F (2018). Effectiveness of body image directed interventions in patients with anorexia nervosa: a systematic review. Int J Eat Disord.

