# Supplementary material for: Exploring multisensory integration of non-naturalistic sounds on body perception in young females with eating disorders symptomatology: a study protocol
Source: J Eat Disord. 2023 Feb 27;11:28. doi: 10.1186/s40337-023-00749-4 (PMC9969697; doi:10.1186/s40337-023-00749-4)

**Supporting information**

**S1 Additional file .** Experimental setup.

Section A: Laboratory room.


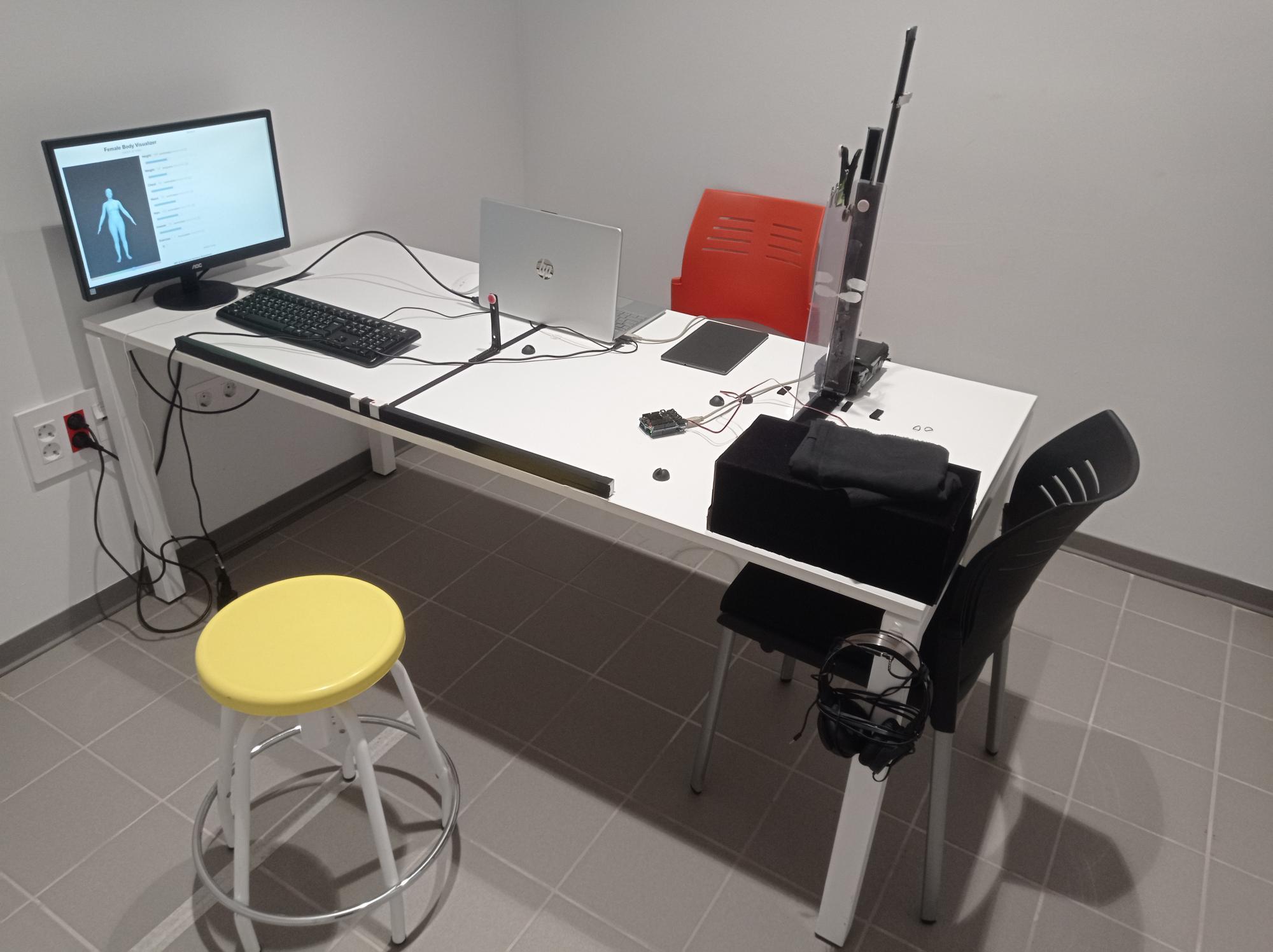


Section B: Anthropometric instruments: Vernier caliper and pediatric stadiometer.


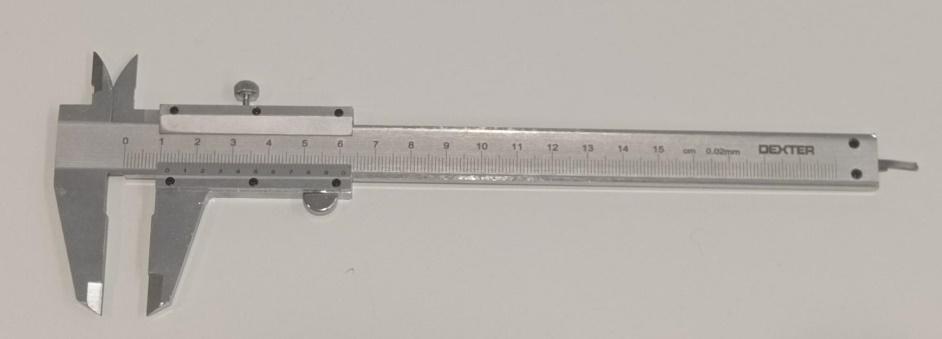


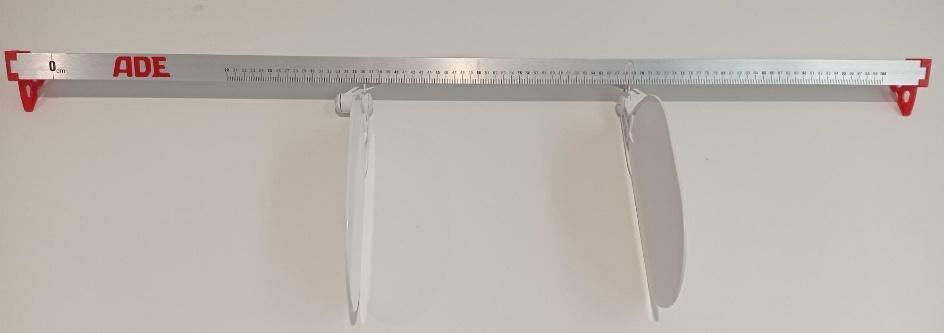

Supplement: Supplementary file 1 — Additional file 1. Experimental setup. Section A: Laboratory room. Section B: Anthropometric instruments: Vernier caliper and pediatric stadiometer. [file 40337_2023_749_MOESM1_ESM.docx]
