# Supplementary material for: Exploring multisensory integration of non-naturalistic sounds on body perception in young females with eating disorders symptomatology: a study protocol
Source: J Eat Disord. 2023 Feb 27;11:28. doi: 10.1186/s40337-023-00749-4 (PMC9969697; doi:10.1186/s40337-023-00749-4)

**Supporting information**

**S2 Additional file .** Experiment 1: Finger. Body feelings questionnaire.

Section A: Spanish version.

*Instrucciones*:

Piense en la experiencia que acaba de tener y marque con un círculo el número que crea que mejor expresa su nivel de acuerdo con las siguientes oraciones.

LEA CADA ÍTEM CUIDADOSAMENTE ANTES DE RESPONDER. Cuando haya decidido su respuesta, marque el número correspondiente.

1. **Mientras escuchaba el sonido sentí que tirar de mi dedo producía el sonido.**


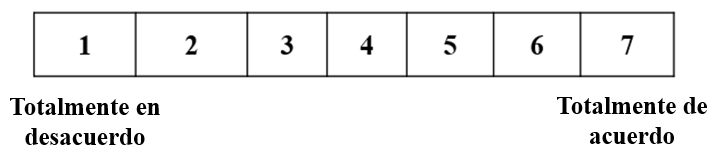


1. **Mientras escuchaba el sonido sentí mi dedo más largo.**


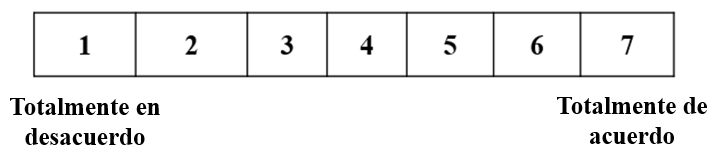


1. **Mientras escuchaba el sonido sentí mi dedo más corto.**


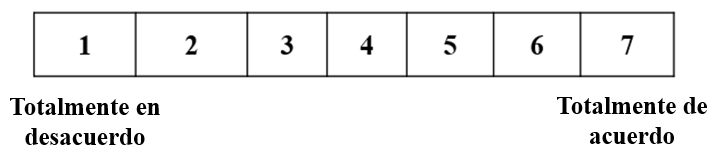


1. **Mientras escuchaba el sonido sentí mi dedo elevarse.**


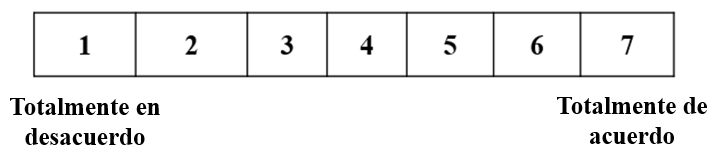


1. **Mientras escuchaba el sonido sentí mi dedo descender.**


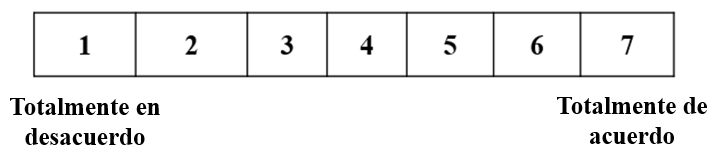


1. **Mientras escuchaba el sonido sentí mi dedo estirarse.**


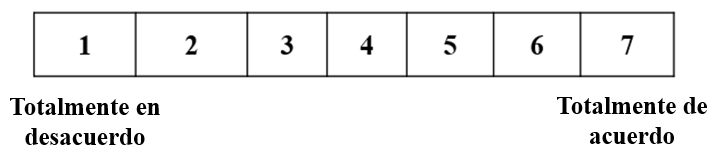


1. **Mientras escuchaba el sonido sentí mi dedo aplastarse.**


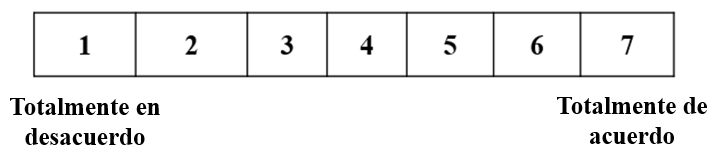


1. **Mientras escuchaba el sonido no sabía decir cómo de largo era mi dedo**


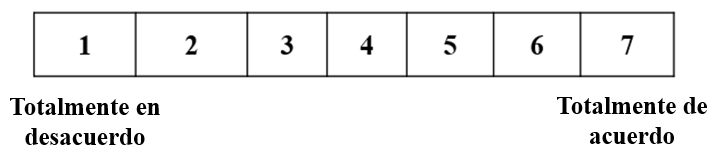


1. **Mientras escuchaba el sonido no podía ubicar la posición de mi nudillo**


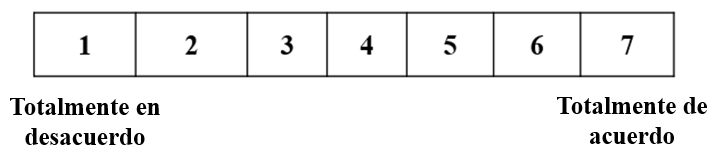


1. **Mientras escuchaba el sonido, no podía ubicar la posición de la punta de mi dedo.**


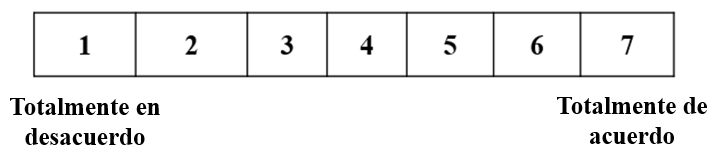


1. **Mientras escuchaba el sonido la sensación que tenía de mi dedo era inesperada.**


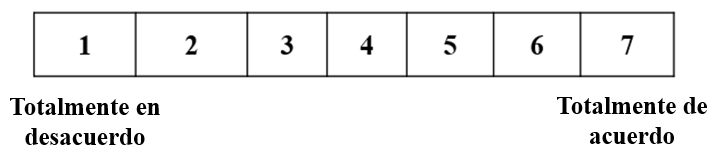


1. **Mientras escuchaba el sonido sentía como si mi dedo no fuera mío.**


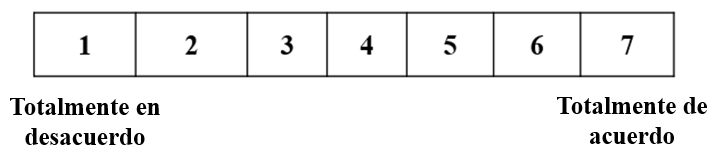


1. **Mientras escuchaba el sonido sentí mi dedo como entumecido.**


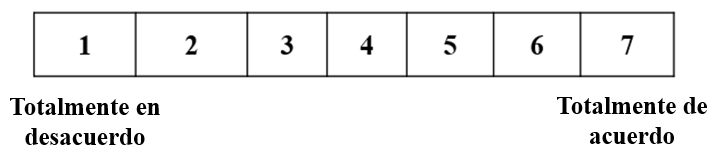


1. **Marque la imagen a continuación que crea que expresa mejor cómo sentiste que era tu dedo al escuchar el sonido.**


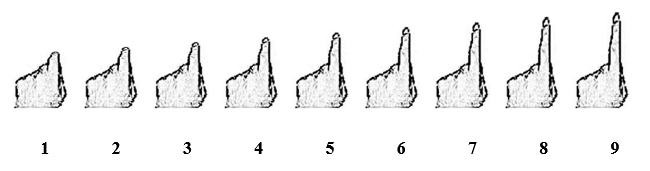


Section B: English version.

*Instructions*:

Think about the experience you just had and circle the number that you think best expresses your level according to the following sentences. READ EACH ITEM CAREFULLY BEFORE ANSWERING. When you have decided on your answer, mark the corresponding number.

1. **I felt pulling on my finger produced the sound.**


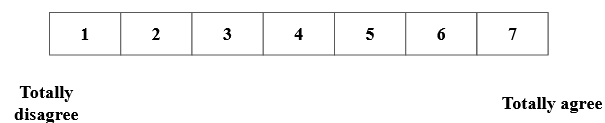


1. **I felt my finger was longer.**


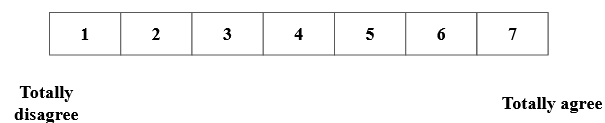


1. **I felt my finger was shorter.**


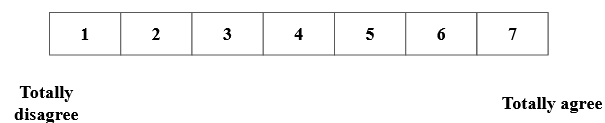


1. **I felt my finger was rising.**


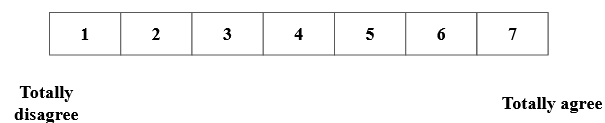


1. **I felt my finger was descending.**


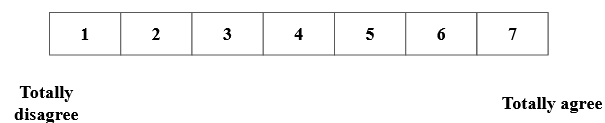


1. **My finger felt stretched.**


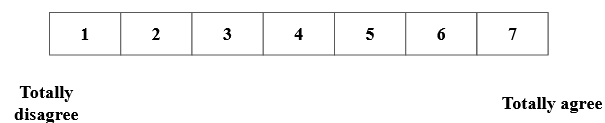


1. **My finger felt squashed.**


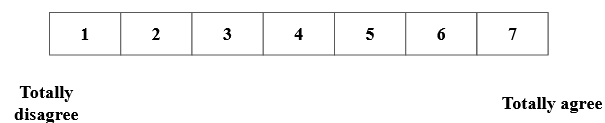


1. **I couldn’t tell how long my finger was.**


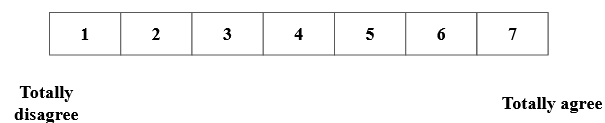


1. **I couldn’t locate the position of my knuckle.**


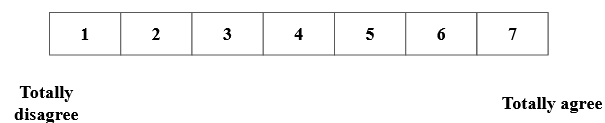


1. **I couldn’t locate the position of my fingertip.**


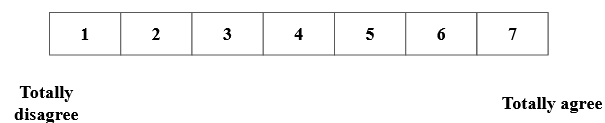


1. **The feeling from my finger was unexpected.**


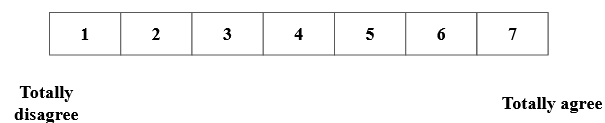


1. **My finger felt like it wasn’t my own.**


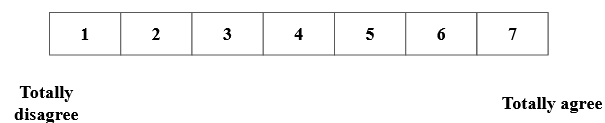


1. **My finger felt numb.**


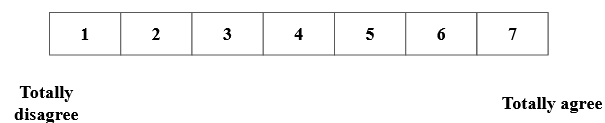


1. **Please cross the picture below that you think better expresses how you felt your finger when listening to the sound.**


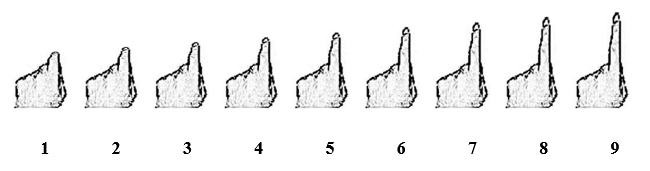

Supplement: Supplementary file 2 — Additional file 2. Experiment 1: Finger. Body feelings questionnaire. Section A: Spanish version. Section B: English version. [file 40337_2023_749_MOESM2_ESM.docx]
