# Supplementary material for: Exploring multisensory integration of non-naturalistic sounds on body perception in young females with eating disorders symptomatology: a study protocol
Source: J Eat Disord. 2023 Feb 27;11:28. doi: 10.1186/s40337-023-00749-4 (PMC9969697; doi:10.1186/s40337-023-00749-4)

**Supporting information**

**S3 Additional file .** Experiment 2: Waist. Body feelings questionnaire.

Section A: Spanish version.

*Instrucciones*:

Piense en la experiencia que acaba de tener y marque con un círculo el número que crea que mejor expresa su nivel de acuerdo con las siguientes oraciones.

LEA CADA ÍTEM CUIDADOSAMENTE ANTES DE RESPONDER. Cuando haya decidido su respuesta, marque el número correspondiente.

1. **Mientras escuchaba el sonido, sentí que tirar de mi cintura producía el sonido.**


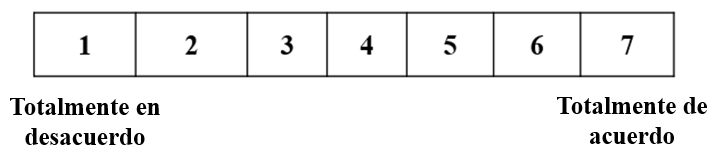


1. **Mientras escuchaba el sonido sentí mi cintura más ancha.**


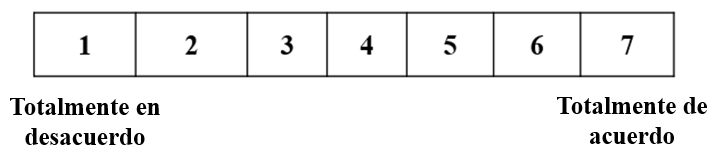


1. **Mientras escuchaba el sonido sentí mi cintura más estrecha.**


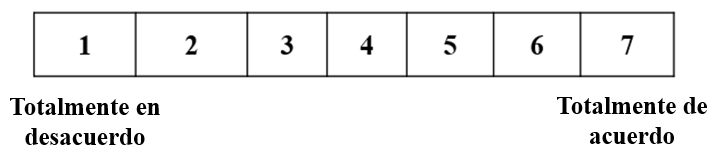


1. **Mientras escuchaba el sonido sentí mi cintura elevarse**


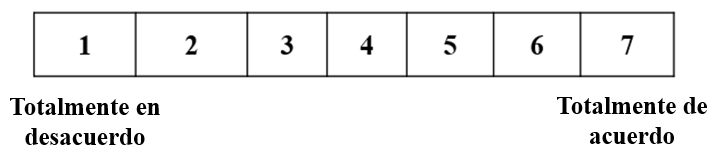


1. **Mientras escuchaba el sonido sentí mí cintura descender.**


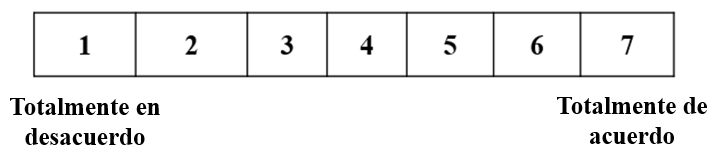


1. **Mientras escuchaba el sonido sentí mi cintura estirarse.**


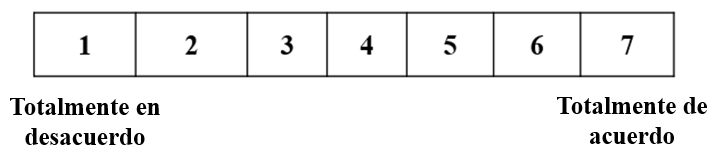


1. **Mientras escuchaba el sonido sentí mi cintura aplastarse.**


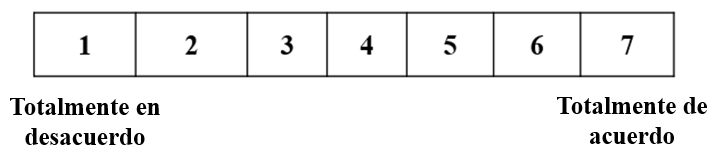


1. **Mientras escuchaba el sonido no sabía decir cuanto medía mi cintura.**


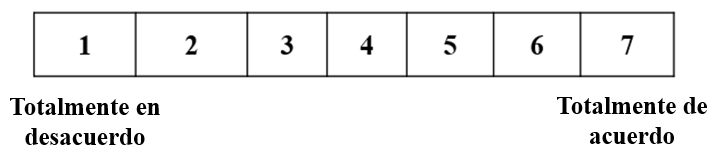


1. **Mientras escuchaba el sonido no podía ubicar los bordes de mi cintura.**


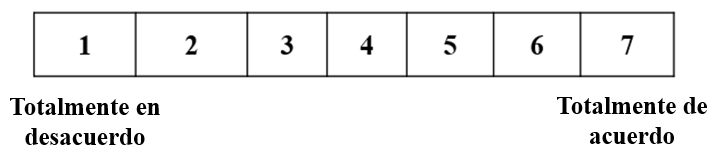


1. **Mientras escuchaba el sonido la sensación que tenía de mi cintura era inesperada.**


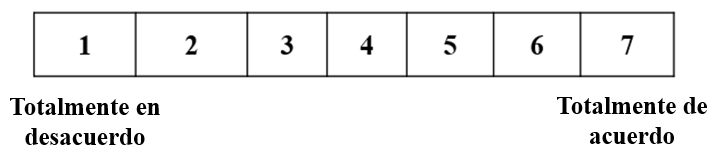


1. **Mientras escuchaba el sonido sentía como si no fuera mía.**


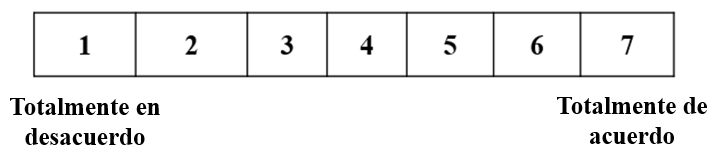


1. **Mientras escuchaba el sonido sentí mi cintura como entumecida.**


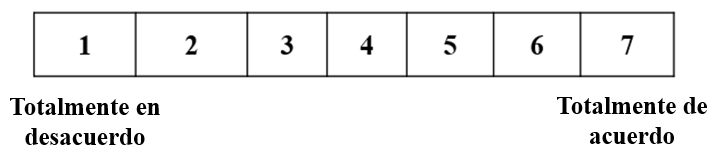


1. **Marque la imagen a continuación que crea que expresa mejor cómo se sentiste que era tu cintura al escuchar el sonido.**


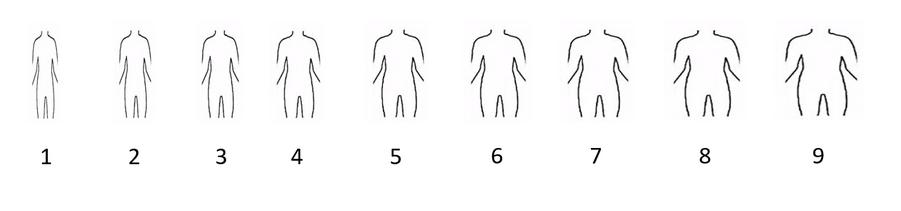


Section B: English version.

Think about the experience you just had and circle the number that you think best expresses your level according to the following sentences. READ EACH ITEM CAREFULLY BEFORE ANSWERING. When you have decided on your answer, mark the corresponding number.

1. **As I listened to the sound I felt that pulling my waist produced the sound.**


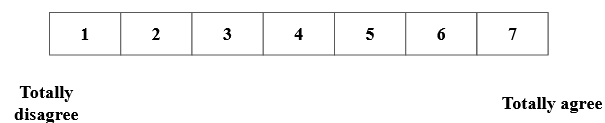


1. **While listening to the sound I felt my waist get wider.**


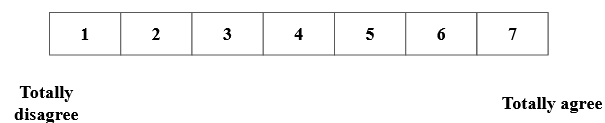


1. **As I listened to the sound, I felt my waist get narrower.**


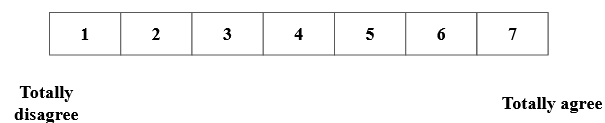


1. **As I listened to the sound I felt my waist rise.**


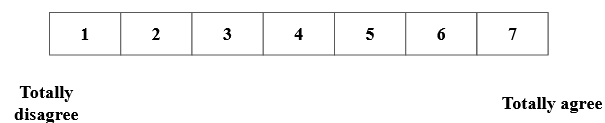


1. **As I listened to the sound I felt my waistline go down.**


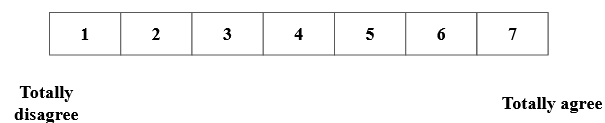


1. **As I listened to the sound I felt my waist stretch.**


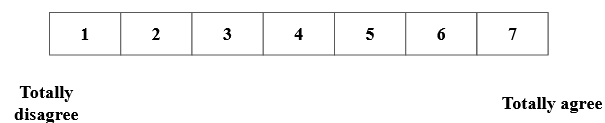


1. **As I listened to the sound I felt my waist flatten.**


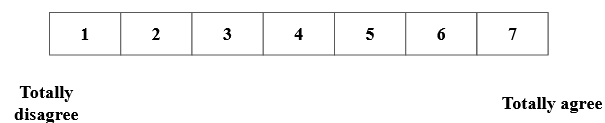


1. **As I listened to the sound I couldn't tell how long my waist was.**


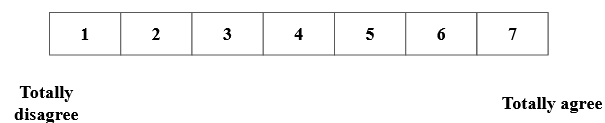


1. **As I listened to the sound I could not locate the edges of my waist.**


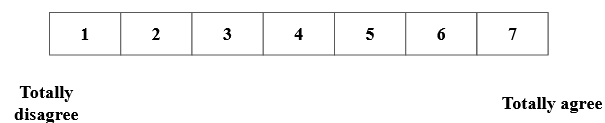


1. **While listening to the sound the feeling I had of my waist was unexpected.**


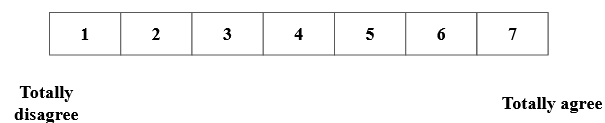


1. **While listening to the sound I felt as if it was not mine.**


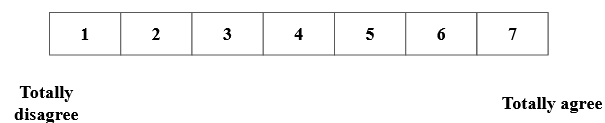


1. **As I listened to the sound my waist felt numb.**


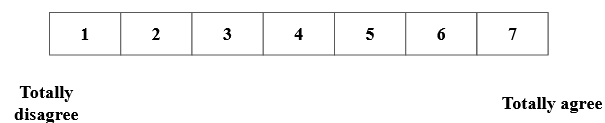


1. **Please cross the picture below that you think better expresses how you felt your waist when listening to the sound.**


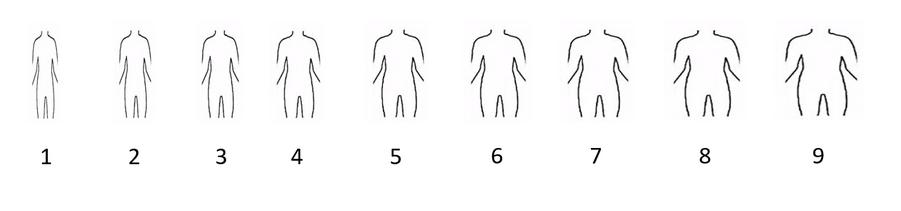

Supplement: Supplementary file 3 — Additional file 3. Experiment 2: Waist. Body feelings questionnaire. Section A: Spanish version. Section B: English version. [file 40337_2023_749_MOESM3_ESM.docx]
